# Closing the gap for drowning prevention across Europe

**DOI:** 10.1016/S2468-2667(22)00193-1

**Published:** 2022-07-24

**Authors:** Amy E Peden, Jonathon Passmore, Ana Catarina Queiroga, Roger Sweeney, Jagnoor Jagnoor

**Affiliations:** a School of Population Health, University of New South Wales, Sydney, NSW, Australia; b Road Safety, Violence and Injury Prevention, WHO Regional office for Europe, Bonn, Germany; c Laboratory for Integrative and Translational Research in Population Health [ITR], Porto, Portugal; d Water Safety Ireland, Galway, Ireland; e The George Institute for Global Health, Newtown, NSW, Australia

Drowning is a preventable cause of premature mortality and was estimated to cause of 236 000 deaths globally in 2019.^1^ In WHO's European region, drowning was estimated to have resulted in the deaths of 19 444 people in 2019, a mortality rate of 2·1 per 100 000.^1^ This rate is lower than the global rate of 3·1 per 100 000 population, and the second lowest of any of the WHO regions, only higher than the rate of 1·8 per 100 000 in WHO's region of the Americas.

However, such regional level data conceals the heterogeneity of drowning burden and context at a country level. Work undertaken to map the epidemiology of unintentional fatal drowning burden across the 53 member states of the WHO European region has revealed extreme variability in drowning rates, ranging from 6·7 per 100 000 people in Latvia to a low of 0·3 per 100 000 people in Iceland and Luxembourg.^1^

Variability is also seen in drowning mortality by sex and age in WHO's 2019 estimates.^1^ Drowning rates for men are as high as 11·3 per 100 000 people in Latvia, 10·2 in per 100 000 people Belarus and 9·8 per 100 000 people in Ukraine. By contrast, drowning rates among women are much lower, with the highest rates observed in Tajikistan (3·7 per 100 000 people), Latvia (2·7 per 100 000 people), and Lithuania (2·4 per 100 000 people). In many countries of the WHO European region, drowning was a top ten leading cause of death for children and people younger than 25 years in 2019 with high rates in Azerbaijan (7·3 per 100 000 infants younger than 1 year) and Kyrgyzstan (5·7 per 100 000 children aged 1–4 years). Conversely, in other countries, drowning risk was greatest among the those older than 75 years, with high recorded drowning rates for people aged 75–79 years in Greece (19·4 per 100 000 people) and people aged 80–84 years in Cyprus (17·5 per 100 000 people).^1^

Although the causes of drowning are numerous and complex, prevention can be achieved through the combination of many simple and feasible actions. Challenges with data availability and robustness make it difficult to quantify the effect of a range of planetary drivers on drowning risk, such as a changing climate,^2^ including heatwave and flooding risk, and migration. Advancing drowning prevention through data collection and well-designed studies, is one of WHO's ten evidence-based interventions and strategies to reduce drowning risk.^3^ However, in many countries in the WHO European region, data on drowning is rarely uniformly collected, regularly updated, or detailed, leading researchers in some countries, such as Portugal, to call for the development of national registries for drowning.^4^

A review of 82 policy documents available in English from 37 of the 53 member states of the WHO European region government's websites highlighted the absence of policies related to drowning. Evidence-informed policy is an aspirational challenge for the field. We mapped WHO recommendations for drowning prevention to policies in the region. Across the WHO European region there were policy relevant documents for pool fencing (n=6), rescue and resuscitation (n=12), disaster management (n=27), waterways (n=8), flotation devices (n=3), swim skills (n=6), watercraft safety (n=13), and preventative activities (n=6). There was also a dearth of policy response to mitigate alcohol-related drowning risk.

Water safety plans were identified for Ireland and the UK. Within the UK an overall water safety plan has been adopted, and specific plans for Scotland and Wales. Although no formal evaluation on the effect of these water safety plans has been done, in 2019, the UK recorded a lower unintentional fatal drowning rate than the European region's average (0·5 per 100 000 people). This reduced rate could be due to the UK's multisectorial approach to drowning prevention, with a commitment to evidence-based interventions derived from the Water Incident Database,^5^ strong crossorganisational collaboration via the National Water Safety Forum, and well resourced rescue services of the Royal National Lifeboat Institute, the Maritime and Coastguard Agency,^6^ and Surf Life Saving Great Britain.

The issue of drowning and its prevention demands evidence informed multisectoral action.^7^ On July 25, 2022, marks the second World Drowning Prevention Day. This year's theme is to “do one thing”^8^ to prevent drowning. Because of the diverse drowning risks present in the European region, we urge stakeholders, researchers, and advocates to consider the following recommendations and take action to reduce drowning risk in the WHO European region. The first recommendation is to capture, analyse, and disseminate national empirical data that quantify drowning burden and identify diverse contexts including place and activity; and data that inform multisectoral response for targeted reduction in drowning deaths and serious injuries. We must also address and close the implementation gap of drowning prevention interventions that are effective through well designed evaluation studies; and map the diversity of sectors and stakeholders with realised or potential interests in drowning prevention at a country and regional level.

We recommend encouraging greater investment in partnerships, joint planning, implementation, and accountability of drowning prevention policies, plans, and strategies; and draw on links between drowning prevention, UN Sustainable Development Goals, and those identified in the UN General Assembly resolution on Drowning Prevention.

Guidelines for media communications must be produced through standardised messaging and appropriate assets that depict positive safe behaviours and national standards for water safety training interventions should be established.

By following these recommendations, we can give these dangers the focus they need and scale up our efforts to reduce this preventable cause of mortality and morbidity.

We declare no competing interests.

The authors affiliated with WHO is alone responsible for the views expressed in this Comment and they do not necessarily represent the decisions or policies of WHO.
